# Assessment of Antibacterial Properties and Cytotoxic Effect of Ethanolic Extracts of Clitoria ternatea and Camellia sinensis Herbal Formulation Against Clinical Pathogens

**DOI:** 10.7759/cureus.58287

**Published:** 2024-04-15

**Authors:** Krithick G Surya, Rajeshkumar Shanmugam, Archana Behera, Mukesh Kumar Dharmalingam Jothinathan

**Affiliations:** 1 Nanobiomedicine Lab, Centre for Global Health Research, Saveetha Medical College and Hospital, Saveetha Institute of Medical and Technical Sciences, Chennai, IND; 2 Centre for Global Health Research, Saveetha Medical College and Hospital, Saveetha Institute of Medical and Technical Sciences, Chennai, IND

**Keywords:** cytotoxicity effect, wound pathogens, urinary tract infection, anti-bacterial activities, ethanolic extract

## Abstract

Aim

The study aims to evaluate the antibacterial properties of ethanolic extracts from *Clitoria ternatea* and *Camellia sinensis* against pathogens causing UTI, wound pathogens, and other clinical bacterial infections and their cytotoxic effects using the brine shrimp lethality assay (BSLA).

Methods

Ethanolic extracts of *C. ternatea* and *C. sinensis* were prepared, and their antibacterial activity was tested against *Staphylococcus aureus*, *Escherichia coli*, *Klebsiella pneumoniae*, *Pseudomonas aeruginosa*, and *Enterococcus faecalis* using the well diffusion method. The cytotoxicity was assessed through the BSLA, determining the LC_50_ values for each extract.

Results

The formulation of both plant extracts exhibited significant antibacterial activity against UTI pathogens, and wound pathogen bacteria showed higher efficacy compared to other studies. The BSLA revealed a dose-dependent increase in toxicity, with *C. ternatea* extracts demonstrating higher cytotoxicity than *C. sinensis*.

Conclusion

The ethanolic extracts of *C. ternatea* and *C. sinensis* possess antibacterial properties against UTI-causing bacteria and show cytotoxic effects in a brine shrimp model. These findings suggest the potential of these plants for developing alternative treatments for UTI. However, further research is necessary to fully understand their safety and efficacy in human subjects.

## Introduction

UTI is a prevalent bacterial infection that impacts a significant number of individuals globally. Uropathogenic microorganisms such as *Escherichia coli* (80-90%), *Staphylococcus aureus* (5-10%), *Pseudomonas aeruginosa*, *Klebsiella pneumoniae*, *Enterococcus faecalis*, and *Proteus mirabilis* are the major causes of UTI [1,2]. UTI is more common in women than in men across all age categories [3]. Up to 50% of women have reported experiencing at least one UTI in their lifetime [4]. Antibiotics are a common component of traditional UTI treatments, but the rise of antibiotic-resistant bacteria has raised serious safety concerns. Antimicrobial natural solutions provide a different strategy for successfully battling UTI [2]. Based on the data, nearly 38,000 individuals were admitted and received medical treatment in 2018 across the world. Despite advancements in burn care in recent decades, wound infections resulting from burns continue to be a significant factor contributing to mortality rates. Recent research has indicated that microbial infections have been responsible for fatalities in 42-65% of burn patients over the last 10 years [5]. The study by Polinarski et al. projected that by the year 2050, multidrug-resistant organisms could result in the deaths of nearly two million individuals across the world [6].

Here, we utilized ethanolic mixed extracts from both *Clitoria ternatea* flowers and *Camellia sinensis* leaves as plant sources. In developing countries, traditional medicine, especially herbal remedies like *C. ternatea* and *C. sinensis*, remains vital, with 80% of the world using plants as primary medicine since ancient times [7]. The tropical leguminous plant *C. ternatea*, butterfly pea, is native to Southeast Asia. Its pharmacological attributes, such as its antioxidant, anti-inflammatory, and antibacterial effects, have been studied in several studies [8,9]. Tea, or *C. sinensis*, is a plant that is widely consumed throughout the world due to its many health advantages. It has a high concentration of polyphenols, such as flavonoids and catechins, which support its anti-inflammatory and anticancer activities [10]. Agbom et al.’s study shows that *C. sinensis* (green tea) methanol and ethyl acetate extracts have bioactive ingredients, and the methanol extract exhibited higher antibacterial activity against the test uropathogenic *E. coli* isolates at lower concentrations than ethyl acetate extract [11]. Likewise, Sharma and Pundir’s study shows that the leaves of *C. sinensis* exhibit antibacterial and antifungal properties, making them a potential alternative to current therapeutic agents with fewer side effects on human skin [12]. Putri and Devientasaria reported that the *C. ternatea* flower (CTF) extract demonstrates antibacterial efficacy against *P. aeruginosa*, with a concentration of 30% exhibiting the most potent inhibitory effect compared to concentrations of 10% and 20% [13].

Further, toxicological studies of the ethanolic extracts were examined by the brine shrimp lethality assay (BSLA). BSLA is a common method to evaluate the toxicity of different compounds. It is utilized in pharmacology as a first screening test to assess the potential cytotoxic impacts of natural substances, such as plant extracts. This study focuses on relevant studies regarding the toxicity evaluation of the ethanolic extract formulations of *C. ternatea *and *C. sinensis*, with an emphasis on the BSLA. *C. sinensis* has the least toxicity on brine shrimp compared to other plants [14,15]. The toxicity levels of the CTF extracts were found to be higher in comparison to the leaf extracts, as demonstrated by the cytotoxicity results observed in the study [15].

The study involves a potential natural therapy option for treating common infections such as UTI caused by uropathogenic bacteria, gastrointestinal infections, antibiotic resistance, and wound pathogenic bacteria using an ethanolic extract formulation of CTF and *C. sinensis* leaf (CSL). Additional investigation is required to pinpoint the precise bioactive compounds accountable for the noted antibacterial effects. Developing alternative treatments is essential to combat antibiotic resistance and improve patient outcomes.

## Materials and methods

The study was conducted at the Nanomedicine Lab, Saveetha Medical College and Hospital, Chennai, India.

Preparation of plant ethanolic extract

**Figure 1:**
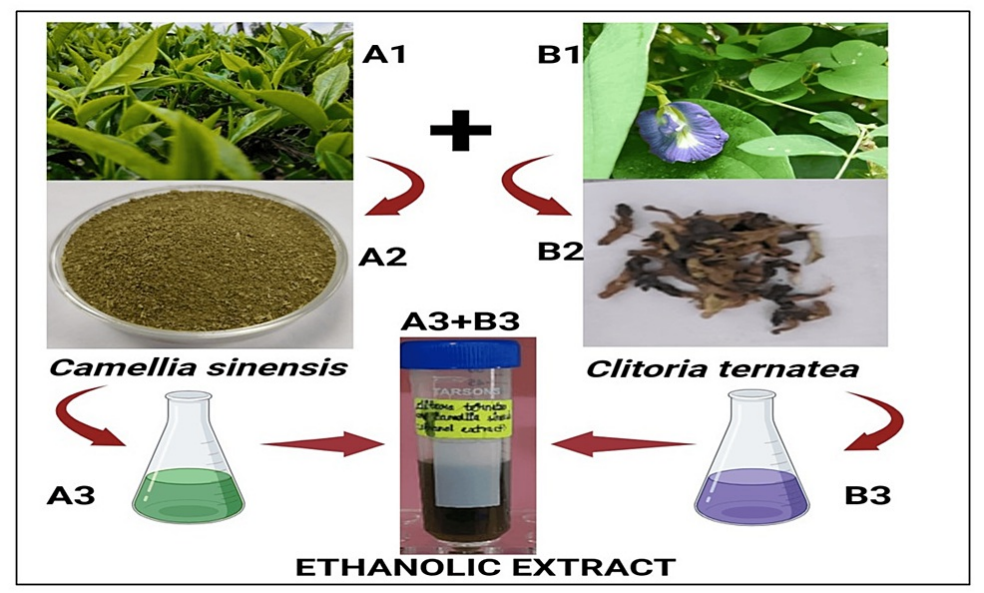
Preparation of herbal formulation by using ethanolic extracts A1: *Camellia sinensis* leaf; A2: *Camellia sinensis *leaf powder; A3: *Camellia sinensis* leaf ethanolic extract; B1: *Clitoria ternatea* flower; B2: dried *Clitoria ternatea *flower; B3: *Clitoria ternatea* flower ethanolic extract; A3+B3: formulation of herbal ethanolic extract

The CTF and CSL were collected, washed thoroughly with distilled water, and shade-dried. The samples were powdered and weighed, and 1.25 g of CTF and 1.25 g of CSL were left to soak in 50 mL of ethanol separately. The extracts were heated using a heating mantle for three to five minutes at 60°C and filtrated using Whatman No. 1 filter paper. After that, ethanol extracts of both samples (CTF and CSL) were kept for further experiments. CTF and CSL ethanol extracts were mixed to prepare the herbal formulation. The herbal formulation was kept closed for 30 minutes in the water bath (70°C) and left overnight in the shaker. After 24 hours, the formulation was then heated again in the water bath (70°C) for an hour and kept for further use. Figure 1 explains the preparation of herbal formulations by using an ethanol solution.

Antibacterial activity

The well diffusion method was used to identify the properties of antibacterials by using CTF and CSL ethanolic extracts. The ethanolic fractions of CTF and CSL were investigated for their antibacterial properties against pathogenic bacteria, including *E. coli*, *K. pneumoniae*, *S. aureus*, *P. aeruginosa*, and *E. faecalis* in this study. The evaluation of antibacterial properties was conducted using the agar well diffusion method as previously described in the literature [16] The bacteria were cultured individually on Mueller-Hinton agar (MHA) at 37°C for 24 hours. Following incubation, the bacterial suspension was spread and plated onto MHA plates. Subsequently, three distinct quantities of CTF and CSL extracts (25 µL, 50 µL, and 100 µL) were introduced into wells (6 mm in diameter) that were taken in the agar plates by using a micropipette. The plates were immediately placed in an incubator set at a temperature of 37°C for 24 hours. Following this, the inhibitory zones were measured in mm. The antibacterial activity assay was conducted in triplicate with the same quantities.

BSLA: cytotoxic effect

A quantity of 2 g of non-iodized salt was measured and mixed in 200 mL of distilled water. A total of 10-12 mL of saline water was added to each six-well plate of the enzyme-linked immunosorbent assay (ELISA). Each well was carefully inoculated with 10 nauplii, using different quantities (5 µL, 10 µL, 20 µL, 40 µL, and 80 µL). The appropriate extract was added based on the desired concentration level. In an incubator, the plates were subsequently placed for 24 hours. The sixth well served as a control, consisting mainly of saline solution and viable nauplii. After 24 hours, the number of live nauplii present in the ELISA plates was examined. The calculation was derived using a precise formula from prior investigations [17].

$\left (Number \hspace{00.2cm} of \hspace{00.2cm} dead \hspace{00.2cm} nauplii \hspace{00.2cm} \div \hspace{00.2cm} Number \hspace{00.2cm} of \hspace{00.2cm} dead \hspace{00.2cm} nauplii \hspace{00.2cm} + \hspace{00.2cm} Number \hspace{00.2cm} of \hspace{00.2cm} Live \hspace{00.2cm} nauplii \right)\times 100$)

Statistical analysis

The experiments for assessing antibacterial activity and cytotoxic effects were conducted in triplicate for each sample. The error bars shown in the graphical representations are expressed as mean ± SD and percentages using the Origin 2018 software. The significance level (p-value) was indicated as <0.05.

## Results

Antibacterial activity

**Figure 2:**
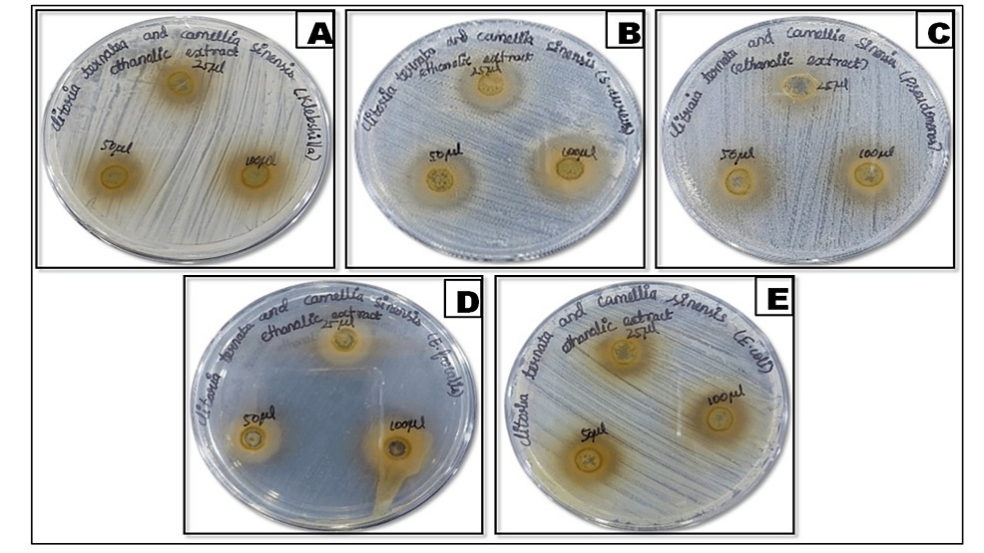
Antibacterial activity of the agar well diffusion method by using ethanolic extracts herbal formulation Herbal formulation: *Clitoria ternatea* flower and *Camellia sinensis* leaf (A) *Klebsiella pneumoniae*, (B) *Staphylococcus aureus*, (C) *Pseudomonas aeruginosa*, (D) *Enterococcus faecalis,* and (E) *Escherichia coli*

**Figure 3:**
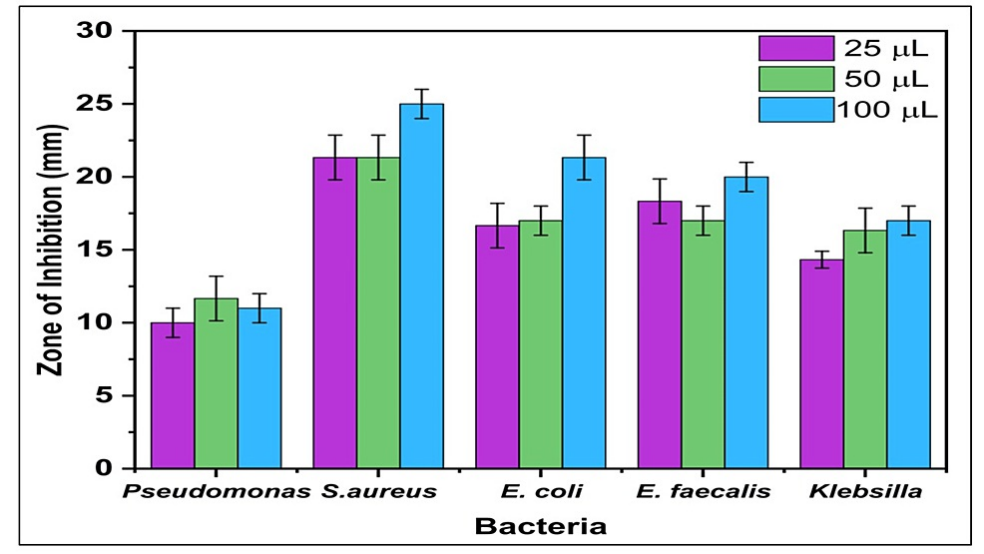
Graphical representation of the antibacterial activity of herbal formulation using ethanolic extracts Herbal formulation: *Clitoria ternatea* flower and *Camellia sinensis* leaf Bacteria: *Pseudomonas aeruginosa*, *Staphylococcus aureus*, *Escherichia coli*, *Enterococcus faecalis*, and *Klebsiella pneumoniae*

To determine the values of the zones of inhibition (ZOIs) for CTF and CSL extracts, the well diffusion method was used. Figure 2 demonstrates the agar well plates of the antibacterial effectiveness of the ethanolic extract of CTF and CSL. The ethanolic extracts of CTF and CSL exhibited the most significant ZOIs against *S. aureus*, measured at doses of 25, 50, and 100 μL, respectively (Figure 2B). Additionally, the following measurements of the ZOIs were seen against *E. faecalis* (Figure 2D), *E. coli* (Figure 2E), *K. pneumoniae *(Figure 2A), and *P. aeruginosa* (Figure 2C) while using different quantities of 25, 50, and 100 μL, respectively. The ethanolic extracts exhibited the greatest ZOIs when applied at a volume of 100 μL, higher than the ZOIs observed with a volume of 25 μL and 50 μL. Figure 3 shows a graphical representation of antibacterial activity, which shows higher ZOIs against *S. aureus*.

Cytotoxic effect

**Figure 4:**
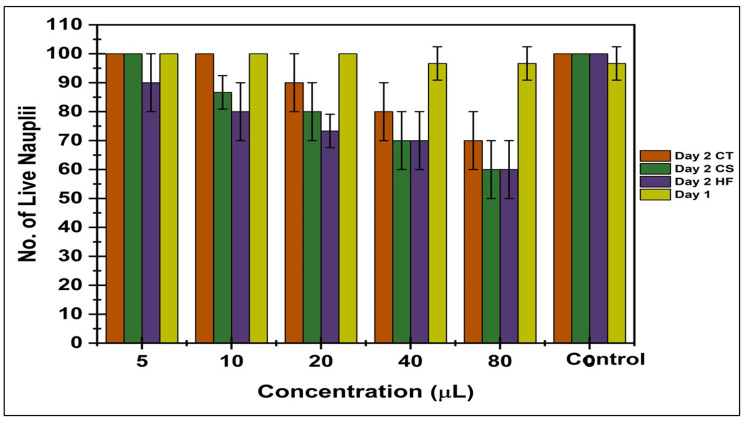
Cytotoxic effect of herbal formulation ethanolic extracts by using BSLA Herbal formulation: *Clitoria ternatea *flower and *Camellia sinensis *leaf BSLA: brine shrimp lethality assay; CT: *Clitoria ternatea*; CS: *Camellia sinensis*; HF, herbal formulation

The cytotoxic effects of CTF and CSL ethanolic extracts were examined using a BSLA. The cytotoxicity of compounds is commonly evaluated using BSLA, which measures their impact on the survival of brine shrimp nauplii. A control group was maintained during this experiment without adding the extracts to determine the percentage of viable nauplii. The findings of the cytotoxicity assessment indicated that the extracts at varied concentrations had diverse impacts on the survival of nauplii. When using 5 μL and 10 μL quantities of extracts, almost 95% of the nauplii survived. Likewise, when using quantities of 20 μL and 40 μL, the extracts could sustain around 80% of the active nauplii. Nevertheless, when the volume was increased to 80 μL, only 70% of the nauplii were alive, as seen in Figure 4.

## Discussion

The discussion of this study finds that multiple research studies have shown that different plant extracts can fight bacteria and be toxic to cells. It mainly examines how these extracts could help treat clinical infections like wounds and the urinary tract. The studies start with general information on how herbal remedies can help with UTI and then focus on specific plants like *C. ternatea* and *C. sinensis* (green tea), showing their potential benefits against these infections.

The report by Das highlights the use of medicinal herbs for preventing or treating UTI, discussing botanicals with established urobactericidal activity, clinical trials on cranberry products, and other natural therapeutics for UTI [18]. This sets the foundation for exploring the antibacterial properties of various plant extracts. Islam et al. found that *C. ternatea* extracts effectively against *E. coli* and *S. aureus* [15]. The study also highlighted molecular docking studies revealing compounds that target antibiotic resistance, suggesting the extract’s potential in combating antibiotic-resistant pathogens. This is complemented by Zulkamal et al., who investigated* C. ternatea *crude extracts against pathogenic bacteria, finding promising antibacterial activity that supports its use as a natural remedy [7]. Rekha et al. also revealed the antibacterial action of *Azadirachta indica*, *Centella asiatica*, and* C. ternatea* extracts and their minimum inhibitory concentration against pathogens like *S. aureus*, *P. aeruginosa*, and *Klebsiella* sp., showing significant pathogen inhibition [19]. A study by Agbom et al. related to *C. sinensis* extracts found that the methanol extract was more effective than the ethyl acetate extract in restoring health markers in infected rats, suggesting green tea’s potential in treating bacterial infections [11]. Kaewkod et al. also discovered that green, oolong, and black tea, along with Thai medicinal plants, especially green tea, and *Garcinia cowa*, inhibit enteric bacteria due to their phenolic and flavonoid content [20].

Additionally, Islam et al. conducted a comparative study on the toxicity of CTF and leaf extracts, finding the flower extracts more toxic to brine shrimp, highlighting differences in cytotoxicity within the plant [15]. Pal et al. also conducted a BSLA on the leaf and stem extracts of *C. ternatea*, noting significant toxicity at various concentrations, with LC_50_ values calculated for leaves and stem extracts [21]. The toxicity test showed that shrimp deaths increased with higher concentrations of green tea leaf extract after 24 hours, linking to the broader discussion on the cytotoxicity of natural extracts [22].

The bioactive compounds present in flavonoids of *C. ternatea* flower, such as anthocyanins, may be attributed to their mechanisms of action such as increased surface hydrophobicity, discharge of intracellular ions, disruption of protein synthesis, free radical scavenging, and inflammation inhibition [23]. Similarly, the flavonol bioactive compounds present in *C. sinensis *leaf may cause cell membrane disruption, inhibition of DNA and protein synthesis, cell envelope biosynthesis inhibition, chelation of metal ions, and multiple mechanisms against Gram-negative bacteria [24].

This organized summary presents a cohesive discussion of the studies’ findings, logically progressing from a general overview of herbal treatments for UTI to specific insights into the antibacterial and cytotoxic properties of particular plant extracts.

Limitations

The study, while revealing the potential of *C. ternatea* and *C. sinensis* ethanolic extracts against clinical pathogenic bacteria, is limited by its narrow bacterial scope, lack of human trials, uncertain safe dosages, and untested effects of variable plant compositions and interactions with other medications.

## Conclusions

This study presents the first report on the antibacterial activity of the *C. sinensis* and *C. ternatea* ethanolic extract against the clinical pathogenic bacteria. *S. aureus* pathogenic bacteria was found to have the highest ZOIs of the formulated tested extracts. The phytochemicals of *C. sinensis* and *C. ternatea* ethanolic extracts showed enhanced antibacterial activity against clinical pathogenic bacteria. The combination of ethanolic extracts from *C. ternatea* and *C. sinensis* exhibits promising antibacterial activity against pathogens that cause UTI, wound infections, and other clinical diseases. This work has documented the toxic effects of the study plants against brine shrimp larvae. Further studies are required to determine the specific toxic components in the extract and to evaluate its efficacy and safety against other target organisms.
